# Single Droplet on Micro Square-Post Patterned Surfaces – Theoretical Model and Numerical Simulation

**DOI:** 10.1038/srep19281

**Published:** 2016-01-18

**Authors:** Y. Q. Zu, Y. Y. Yan

**Affiliations:** 1 Department of Mechanics and Engineering Science, Fudan University, Shanghai, 200433, P.R. China; 2 Faculty of Engineering, University of Nottingham, University Park, Nottingham NG7 2RD, UK

## Abstract

In this study, the wetting behaviors of single droplet on a micro square-post patterned surface with different geometrical parameters are investigated theoretically and numerically. A theoretical model is proposed for the prediction of wetting transition from the Cassie to Wenzel regimes. In addition, due to the limitation of theoretical method, a numerical simulation is performed, which helps get a view of dynamic contact lines, detailed velocity fields, etc., even if the droplet size is comparable with the scale of the surface micro-structures. It is found that the numerical results of the liquid drop behaviours on the square-post patterned surface are in good agreement with the predicted values by the theoretical model.

The study of improving surface wettability is an important subject of physics and can essentially influence a lot of cutting-edge topics in engineering such as textile^1^, micro/nano- fluidics^2^,^3^,^4^,^5^, film coating^6^ and tribology^7^. The effect of surface chemistry^8^,^9^,^10^,^11^,^12^,^13^ and topography^14^,^15^,^16^,^17^,^18^,^19^,^20^,^21^ on the wettability of surfaces has been intensively investigated over the past decades, and a remarkable progress has been achieved. It has been recognised that an appropriate modification on surface chemistry or structure can improve and help optimize surface wettability. The chemical modification of surface alone can typically make water contact angles up to 120°; and to reach the extreme values of contact angles approaching approximately up to 180°, a modification on surface structure has to be utilized^22^,^23^. Depending on the original chemical properties of surfaces, micro-roughness may make surfaces either more hydrophilic or more hydrophobic.

So far, the effects of surface roughness on wettability have been studied theoretically for several decades^24^,^25^,^26^,^27^. The pioneering work was carried out by Wenzel^28^ and Cassie-Baxter^29^ who proposed Wenzel equation 27 and Cassie–Baxter equation^29^ on the basis of Young’s law^30^ to predict the wetting behaviors of a droplet. However, such equations are not sufficient to thoroughly explain the mechanisms of wetting phenomena, although they are still necessary^23^. Numerical investigations have been carried out by several researchers based on either molecular dynamics method^31^,^32^,^33^,^34^,^35^ or the lattice Boltzmann method^36^,^37^,^38^,^39^ to study the mechanism of wetting transitions. A comprehensive review on the progress in understanding wetting transitions on rough surfaces have recently been completed by Bormashenko^40^. Very recent studies have focused on manipulating roughness to fabricate surfaces with superhydrophobicity or superhydrophilicity. However, for the same roughness value, different surface geometries can exhibit completely different wetting behaviour. As typical surface geometries, surfaces with pillars (post or spike) patterned have been extensively investigated theoretically and experimentally^20^,^24^,^25^,^26^,^41^,^42^,^43^,^44^,^45^. It is argued whether or not all geometrical parameters of pillars, such as height, spacing, and cross sections, individually play an important role in determining wetting behaviour. This indicates that it is far from enough to only consider the value of surface roughness.

To simulate the behaviours of two-phase droplets with relatively high density ratio, a lattice Boltzmann method (LBM) was developed for studying both the static and dynamic behaviours of droplets on micro-roughness surfaces^46^. The method has already shown its capability in dealing with droplet behaviours on chemically homogeneous and heterogeneous surfaces with large density ratio up to 1000. In general, the LBM has demonstrated a significant potential and broad applicability with many computational advantages, which includes the parallelism of algorithm and the simplicity of programming^47^,^48^,^49^,^50^,^51^,^52^,^53^,^54^,^55^.

In this study, the wetting phenomena of a droplet spreading on a micro square-post patterned surface are analyzed theoretically and simulated numerically; the effects of surface geometrical parameters on the wetting properties are also discussed.

## Results

**Figure 1:**
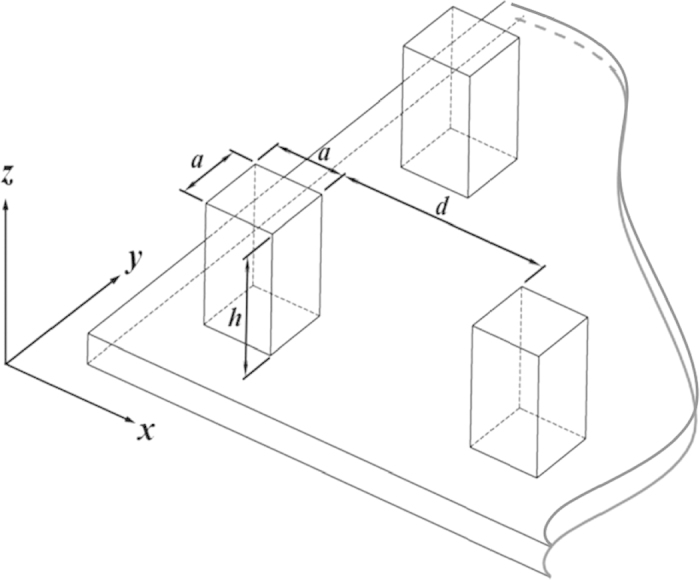
Sketch of square-post patterned surface.

We analyze the behaviours of a droplet on the substrate patterned by structured micro-square posts, as shown in Fig. 1; where *h, a* and *d* are the height, width and spacing of the posts, respectively. The theoretical analysis is based on the assumption that the size of the micro posts is much smaller than that of the droplet. Thus, the wetting properties from a single unit of the surface with periodical square-post pattern are studied.

### Background

**Figure 2:**
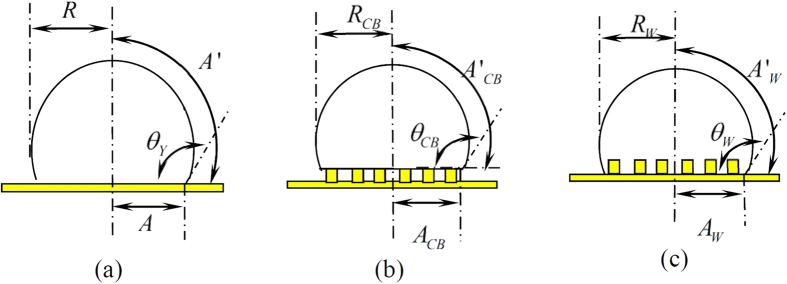
Cross-section view of the droplet on the surface: **(a)** droplet on flat surface (**b**) droplet in Cassie state (c) droplet in Wenzel state.

Figure 2(a) shows the cross-section of the droplet on a flat partial wetting wall, when the relation of contact angle *θ*_*Y*_ is governed by Young’s equation^30^


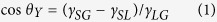


where, *θ*_*Y*_ is named as Young’s angle; *γ*_*LG*_, *γ*_*SG*_ and *γ*_*SL*_ are the surface tension forces of liquid-gas, solid-gas and solid-liquid, respectively.

Correspondingly, the equilibrium net surface energy, Ψ_*Y*_, can be given by^56^,^57^,^58^


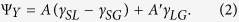


where, *A* and *A*′, respectively, denotes the area of solid-liquid and liquid-gas interfaces.

Similarly, as shown in Fig. 2(b), when the droplet in Cassie state (composite state), the contact angle, surface tension and the equilibrium net surface energy are related by


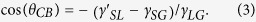



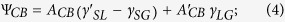


where, *γ*′_*SL*_ is the effective energy per unit area of solid-liquid interface, *A*_*CB*_ the area of drop bottom projected on horizontal plane and *A*′_*CB*_ the area of the surface of drop contacting with bulk gas phase.

While, as shown in Fig. 2(c), for the droplet in Wenzel state the contact angle and the equilibrium net energy, respectively, can be valuated from the following equations:


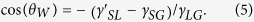



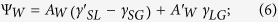


It should be noted that, from microscopic point of view, as shown in Fig. 2(b,c), the drop bottom has many equilibrium states, and each of them has a free energy satisfying Eq. (2). Thus, Ψ_*CB*_ and Ψ_*W*_ can be written as,


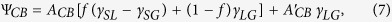


and


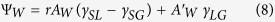


where, *f* is the fraction of the contact area referred to as the ratio of the total area of the liquid-solid interface projected on the horizontal plane with respect to the total area of drop bottom projected on the plane; *r* corresponds to the “roughness parameter”, also referred to as the roughness area ratio of the actual surface with respect to the geometric surface.

Combining Eq. (1), (3), (4) and (7) leads to a Cassie-Baxter’s equation 29.


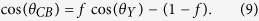


While, combining Eq. (1), (5), (6) and (8) results in a Wenzel’s equation 28.


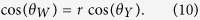


Obviously, depending on the Young’s angle *θ*_*Y*_, micro-roughness may make surfaces either more hydrophilic or more hydrophobic.

### Wenzel’s angle

Let *α* = *d*/*a* and *β* = *h*/*a*, the effects of surface geometrical parameters *α* and *β* on the wetting properties can be verified. When the droplet keeps in Wenzel state, according to Wenzel’s equation, the apparent contact angle *θ*_*W*_ is given by


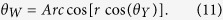


where, the surface roughness parameter *r* is given by


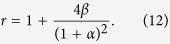


It can be noted from Eq. (11) that the increase of parameter *α*, i.e. d/a, may decrease Wenzel’s angle on a hydrophobic surface, but will increase the angle on a hydrophilic surface. While the increase of *β*, i.e. h/a, may make the hydrophilic surface to be more hydrophilic and hydrophobic surface to be more hydrophobic.

### Cassie-Baxter’s angle

If the droplet is in Cassie state, the apparent contact angle *θ*_*CB*_ can be evaluated with Cassie and Baxter’s equation as,


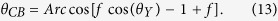


where, the fraction of the contact area, *f*, is given by


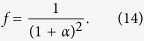


Eqs (13 and 14) indicate that, depending on the original chemical properties of surfaces, lager contact angles can be obtained by increasing the value of *α* if the droplet remains in Cassie state.

### Wetting transition

Form thermodynamic point of view, the droplet always tends to be in a lower energy state. Thus, a threshold Young’s angle exists. Bico *et al*.^59^ has suggested a critical Young’s angle between Wenzel state and Cassie state, when Ψ_*CB*_ = Ψ_*W*_,


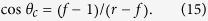


For a flat surface, if the contact angle *θ*_*Y*_ is larger than the critical Young’s angle *θ*_*c*_, the droplet will stay in a stable Cassie state. For *θ*_*Y*_ < *θ*_*c*_, on the other hand, the droplet will exist in Wenzel state. However, it has been found that there is an energy barrier^56^ between the Cassie and Wenzel regimes, so that the droplet could exist in at a metastable wetting state, which may affect the threshold contact angle *θ*_*c*_.

**Figure 3:**
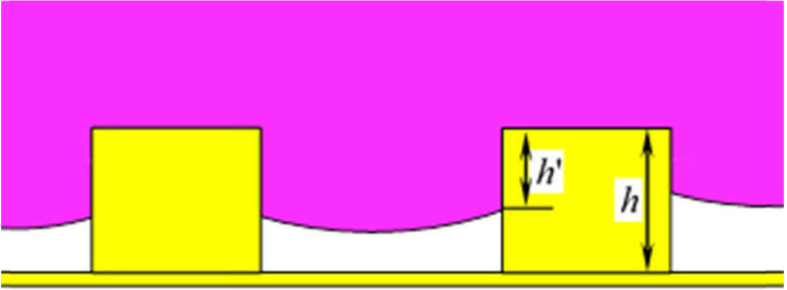
Cross-section view of the droplet in transition from Cassie to Wenzel state.

As mentioned above, a droplet on the roughness surface could exist in a metastable wetting state that does not belong to the Cassie or the Wenzel regimes, as shown in Fig. 3. Once an additional stimulus is applied, a complete transition can take place. It is noted that, from microscopic viewpoint, Eq. (2) is still available even under the metastable equilibrium states, which allows us to predict the apparent contact angles when droplets are in metastable states.

It is noted that the inter-post liquid-gas interface need to go down a depth of *h* to complete a transition from Cassie state to Wenzel state, as shown in Fig. 3. According to Eq. (2), an extreme free energy can be expressed as


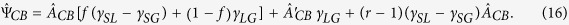


Thus, the energy barrier from Cassie to Wenzel regimes can be evaluated as


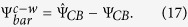


Note that Eq. (16) gives a critical contact angle as,


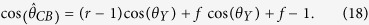


If the drop has a free energy, 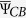, satisfying


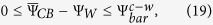


then it could stay in the metastable Cassie state. Otherwise, the droplet either exists in stable Wenzel state or in stable Cassie state. Let 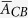, 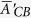 and 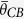 to be the surface area of the metastable droplet, projected area of the metastable droplet bottom and apparent contact angle respectively, then


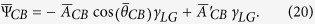


Substituting Eq. (2), (8) and (20) into the left part of in equation (19) gives


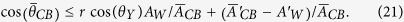


While, substituting Eq. (2), (7), (8), (16), (17) and (20) into right part of inequality (19), we have


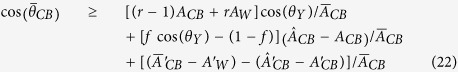


Assuming that the drop has constant volume, the original shape of the droplet is spherical with radius *r*_*s*_ and that the shape of equilibrium droplet after contacting with the surface is a spherical cap with radius *R, R*_*CB*_ or *R*_*W*_ as shown in Fig. 2, the values of *A*_*CB*_, *A*′_*CB*_, *A*_*W*_, *A*′_*W*_, 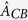, 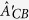, 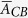, 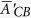 in the inequalities (21) and (22) can then be calculated as


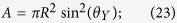



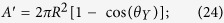



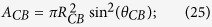



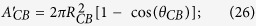



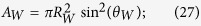



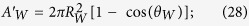



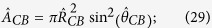



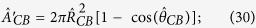



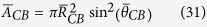



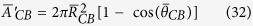


where


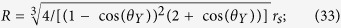



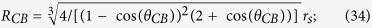



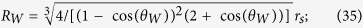



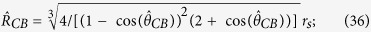



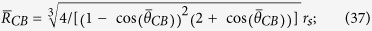


Inequalities (21) and (22) are both implicit inequalities of 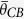 and can be solved numerically using dichotomy algorithm. Neglecting the changes of droplet surface area and projected area of droplet bottom during the transition progress, inequalities (21) and (22) can be simplified to an implicit equation as


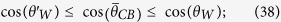


where,


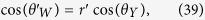


and *r*′ is a virtual roughness parameter given by


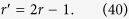


When *θ*_*Y*_ ≥ 90°, the solution of inequality (38) exists.

Depending on the assumption that Eq. (3) is still available during the wetting transition progress from Cassie to Wenzel state, i.e. 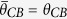, then the left part of inequality (38) gives a modified critical Young’s angle as


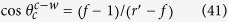


**Figure 4:**
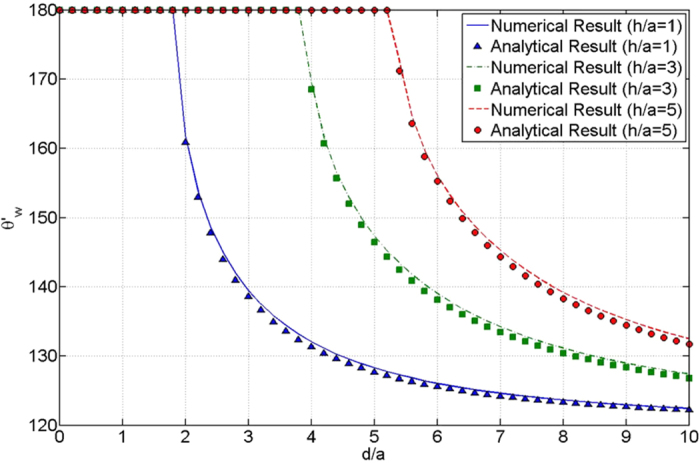
Comparison of numerical results and analytical results of *θ*′_*W*_.

For *a* = 50 *μm, R* = 1000 *μm* and *θ*_*Y*_ = 120°, Fig. 4 shows the comparison of *θ*′_*W*_ obtained by numerical solution of Eq. (22) and analytical solution of the left part of Eq. (38). The good agreement appears. This indicates that the changes of droplet surface area and projected area of droplet bottom during the wetting transition process are negligible.

Briefly, depending on the original chemical properties of surfaces where *θ*_*Y*_ ≥ 90°, droplet could stay in a stable Cassie state if *θ*_*CB*_ < *θ*_*W*_; and if *θ*_*W*_ ≤ *θ*_*CB*_ ≤ *θ*′_*W*_, a transition from Cassie to Wenzel state can take place; else if *θ*_*CB*_ > *θ*′_*W*_, the droplet can complete the transition to a stable Wenzel state. It should be pointed out that, although the present theoretical model is effective to predict the wetting transition from the Cassie to Wenzel regimes, the kinetics of the energy barrier separating the Cassie and Wenzel wetting states should be much more complicated^60^.

### Numerical simulation

As it was mentioned before, the above theoretical analysis is limited to the conditions that the scale of surface micro-structures is much smaller than that of the droplet and is not able to predict the dynamic contact angle. Therefore, a numerical simulation on wetting behaviour of a water droplet with a size comparable with the scale of square-posts on the surface is carried out by extending the LBM^46^,^61^.

**Figure 5:**
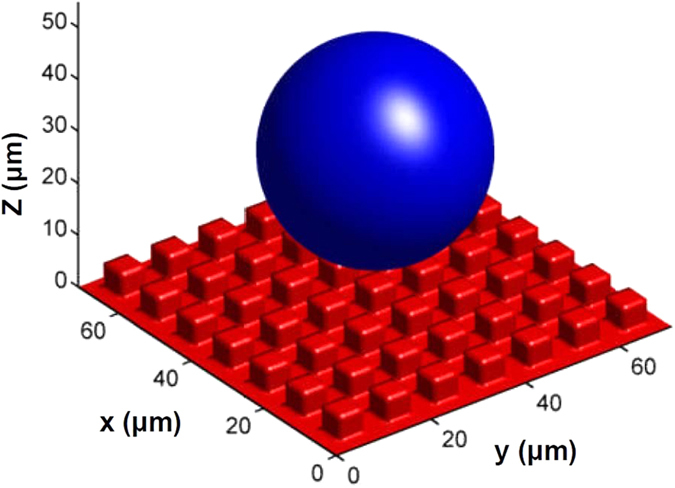
Initial droplet shape and location.

As shown in Fig. 5, a spherical water drop with an initial velocity of 0.01 m/s falls down to the square-post patterned surface; the original radius of the droplet is *r*_*s*_ = 15 *μm*; the initial vertical distance from the droplet center to the top of post is 20 *μm*. Naturally, the densities of water and air are set at *ρ*_*L*_ = 1 × 10^3^ *kgm*^−3^, *ρ*_*G*_ = 1.204 *kgm*^−3^, the dynamic viscosities of them are *μ*_*L*_ = 1 × 10^−3^ *kgm*^−1^*s*^−1^, *μ*_*G*_ = 1.9 × 10^−5^ *kgm*^−1^*s*^−1^, liquid-gas surface tension is *γ*_*LG*_ = 7 × 10^−2^ *J* ⋅ *m*^−2^ and the gravitational acceleration *g* = 9.8 *ms*^−2^.

A cubic uniform grid is meshed and the grid independence certification is performed to select appropriate dimensional size of lattice spacing as Δ*x* = 1 *μm*. For *a* = *d* = *h* = 5 *μm* and *θ*_*Y*_ = 120°. The obtained numerical results will be compared with those of theoretical prediction. It should be pointed out that, with a decrease of droplet size, the contribution of line tension free energy becomes increasingly important^62^,^63^,^64^,^65^. The effects of Line tension (i.e., the excess free energy of a solid-liquid-vapour system per unit length of the contact line) may not be negligible for droplet with a smaller size comparable with the scale of surface micro-structures. Therefore, the line tension effect needs to be checked in this case to ensure that the Cassie-Baxter equation (9) is still available. To take into account the line tension effects, a modified Cassie-Baxter’s equation was proposed by Wong and Ho^66^ as


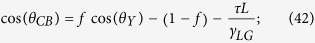


where, *τ* is the line tension, *L* is the length of three-phase contact line per unit area. For the square-post patterned surfaces, as shown in Fig. 1, *L* = 4*a*/(*a* + *d*)^2^. In this particular case, *L* = 1/*a* = 2 × 10^5^ *m*^−1^, *γ*_*LG*_ = 7 × 10^−2^ *J* ⋅ *m*^−2^. According to the high resolution scanning microscopy measurements^67^,^68^, contact line tensions are in the order of 10^−11^ to 10^−10^ *J* ⋅ *m*^−1^. Thus, the third term on the right hand side of Eq. (42) should be on the order of 10^−5^ to 10^−4^, which has limited effect on apparent contact angle. This means that, under the present condition, Cassie-Baxter’s equation (9) is available. However, when the droplet size approximates to be the scale of the surface micro-structures, the error caused by the approximation of the fraction of the contact area *f* with Eq. (14) becomes lager since the equation is obtained on the basis of the assumption that the size of micro posts is much smaller than that of the droplet. To avoid this error, in the present comparison study, the parameter *f* used in theoretical prediction is set to be the same as that in LBM numerical simulation rather than the value given by Eq. (14).

**Figure 6:**
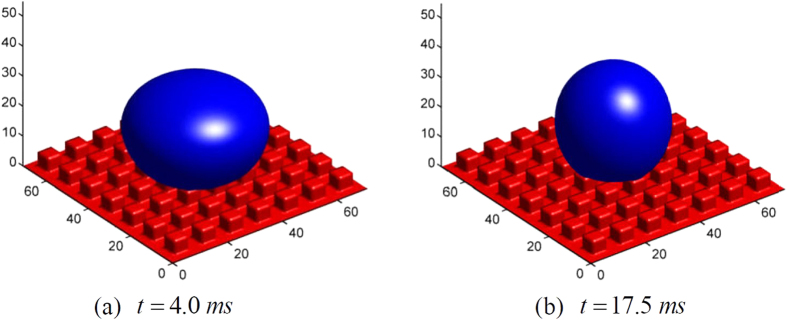
3D view of the droplet shapes.

**Figure 7:**
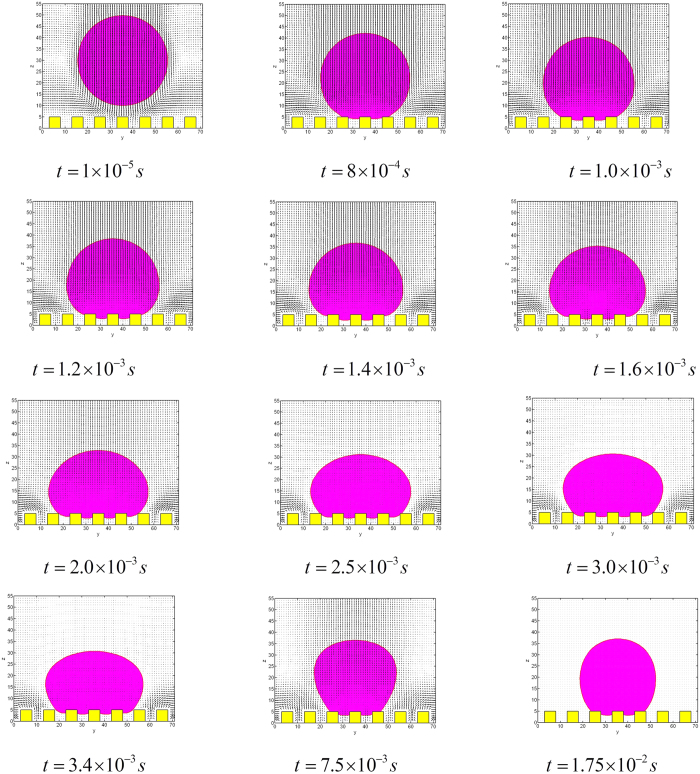
Dynamic interface shapes and the velocity field at the x-z cross-section.

Figure 6 shows the dimensional shape of the droplet when *t* = 2 × 10^−3^ *s* and *t* = 1.75 × 10^−2^ *s*; and the development with time of three phase contact line and the corresponding velocity field are given in Fig. 7. It can be seen that, in spite of an initial momentum, the droplet is still able to stay at a Cassie state; moreover, the movement can finally reaches an equilibrium state when *t* ≈ 17.5 *ms*. The obtained equilibrium contact angle of LBM simulation is 153.16°, for *f* = 0.259; while the theoretical prediction using Cassie-Baxter’s equation for *f* = 0.259 gives a slightly lower value of *θ*_*CB*_ = 150.52°.

**Figure 8:**
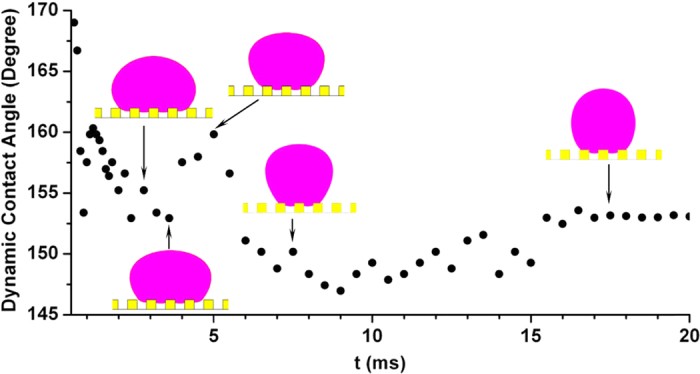
Dynamic contact angles.

The corresponding evolution of dynamic contact angle measured at the middle x-z cross section is shown in Fig. 8. It can be found clearly that there is an oscillation of apparent contact angle with higher frequency at the initial stage of the evolution due to the impingement of the droplet on the solid wall. Also, there is still an oscillation of contact angle with smaller amplitude caused by the interaction of droplet and the post corner of the solid surface.

## Discussion

**Figure 9:**
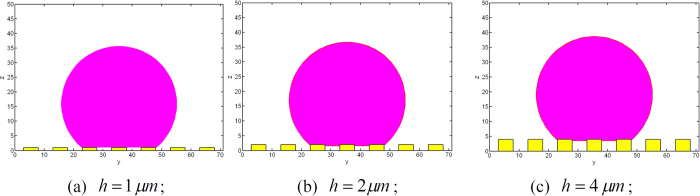
Initial states of the droplets.

To check the effects of surface roughness on wetting transition, equilibrium contact angles of spherical water droplet with *r*_*s*_ = 20 *μm* on surfaces with the same values of width and spacing of the posts, *a* and *d*, but different height of the posts, *h*, are examined. The roughness parameters and chemical property used in the simulation are *a* = *d* = 5 *μm, h* = 1, 2, 4 *μm* and *θ*_*Y*_ = 120°. Initially, the droplet is set at a Cassie state with velocity of zero as shown in Fig. 9.

**Figure 10:**
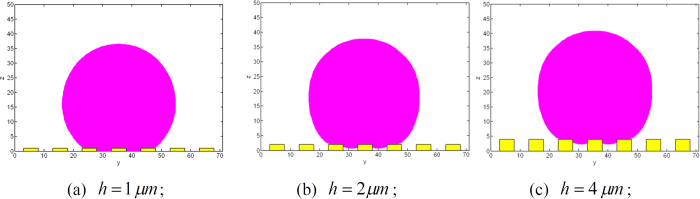
Equilibrium states of the droplets.

As shown in Fig. 10, when reaches the equilibrium state, the droplet on the surface with *h* = 2 and 4 *μm*, respectively, is at Cassie state; while, the droplet on the surface with *h* = 1 *μm* has been transferred to Wenzel state.

**Figure 11:**
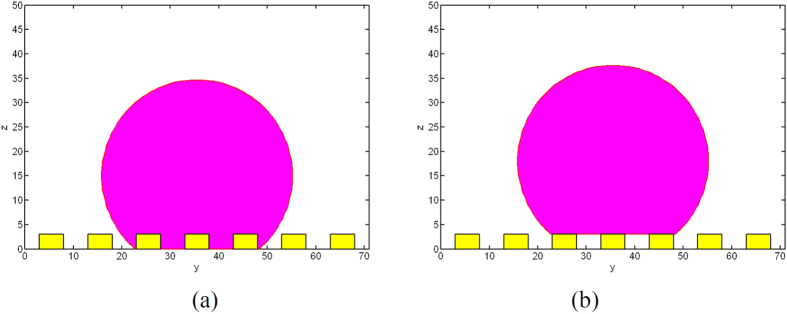
Initial states of the droplets, *h* = 3 *μm*.

**Figure 12:**
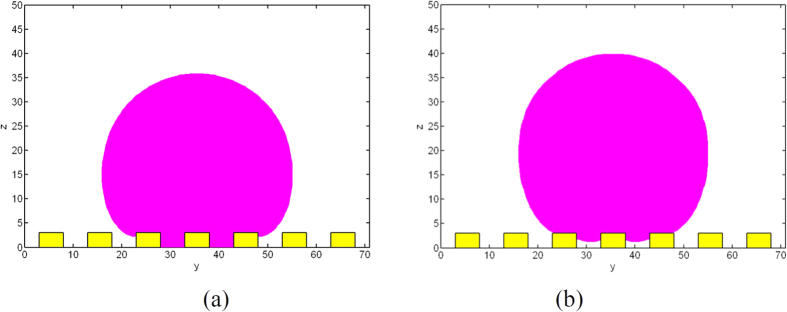
Equilibrium states of the droplets, *h* = 3 *μm.*

To check the effects of energy barrier, a liquid drop of radius *r*_*s*_ = 20 *μm* on the surface with property of *a* = *d* = 5 *μm, h* = 3 *μm* and *θ*_*Y*_ = 120° is simulated (Fig. 11). The droplet initially at both Wenzel and Cassie states are considered. For the same surface property, different equilibrium states are obtained due to the energy barrier. As shown in Fig. 12, the droplet initially at Wenzel or Cassie states can finally reach the equilibrium Wenzel and Cassie states, respectively.

**Figure 13:**
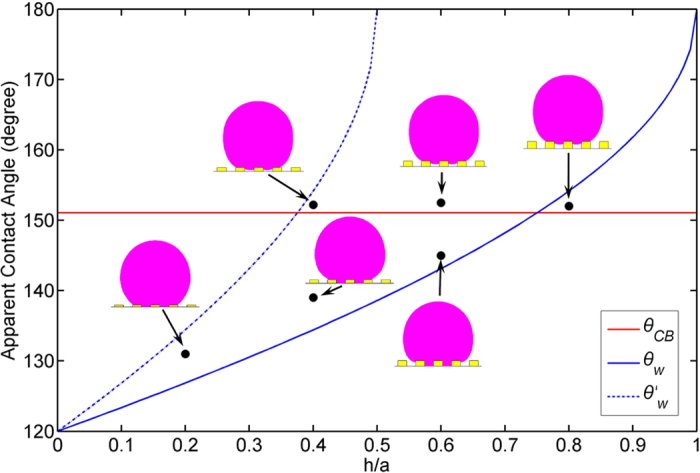
Comparison of wetting transition between analytical and numerical results.

To validate the proposed theoretical model of wetting transition, a comparison between the analytical and numerical results, as shown in Fig. 13, is performed under the condition that the surface is square-post patterned with *θ*_*Y*_ = 120°, *a* = *b* = 5 *μm*, and *h* increases form 0 to 5 *μm*. According to the proposed model in this paper, a critical value of *h*/*a* = 0.38 is obtained by calculating *θ*_*CB*_ = *θ*′_*W*_. It can be seen from the figure that the proposed wetting transition model can give more accurate critical value of *h*/*a* than traditional model^59^ which calculates critical *h*/*a* on the basis of the solution of *θ*_*CB*_ = *θ*_*W*_ because the energy barrier may decrease the critical value of *h*/*a* for wetting transition. It should be pointed out that the present theoretical perdition still uses Eqs (12 and 14) to calculate surface roughness parameter *r* and the fraction of the contact area *f*. Thus, one of reasons causing the differences in apparent contact angle between the numerical and theoretical results should be an approximation of *r* and *f* with Eqs (12 and 14) when the droplet size is comparable with the scale of the surface micro-structures. For the cases in the present study, the differences in *r* and *f* between the theoretical and numerical results are up to 3.3% and 3.5%, respectively.

In this study, the wettability of micro-roughness surfaces, especially of the square-post patterned surfaces is studied theoretically and numerically. A theoretical model of wetting transition is proposed on the basis of the concept of net free energy and energy barrier. Furthermore, a numerical simulation using LBM is carried out successfully to show surface wetting properties, droplet shapes evolution, dynamic contact angle, and corresponding velocity fields. The comparison of the wetting behavior on the square-post patterned surface shows that the predicted values by the proposed theoretical model are in good agreement with the numerical results. As current simulation is to validate the proposed theoretical model of wetting transition, only the surfaces patterned by square posts with constant spacing and height are considered. It is known that each geometrical parameter of the posts such as the height, width and spacing, etc., plays an important role in determining wetting behavior; and this will be considered in our future work.

## Methods

**Figure 14:**
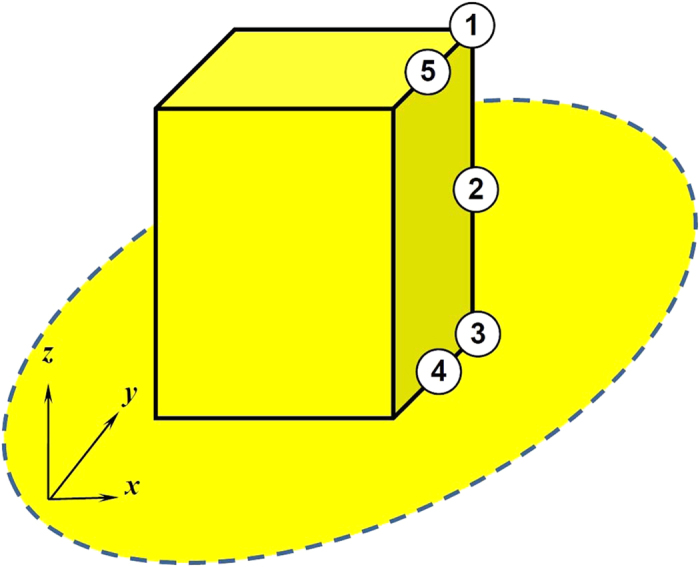
Label of positions of the square post.

In the present study, a lattice Boltzmann method^46^ of Yan & Zu is extended to simulate the wetting behaviors of a droplet spreading on micro-roughness surfaces. In order to implement the wetting boundary condition on solid wall of a liquid-gas system, the order parameter, *ϕ*, in LBM, derivative at the wall boundaries must be given to control the contact angle. The boundary treatment of two phase flow on flat partial wetting surfaces can be given by^46^


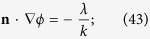


where, **n** is the local normal direction of the wall pointing to the fluid, *λ* a coefficient of wetting potential and *k* the surface tension coefficient. However, for the square-post patterned surface, **n** does not exist at the intersection of two or three orthogonal planes. In the present study, it is artificially defined to have the same angle to the two or three planes^61^. As shown in Fig. 14, at the top corners of the square posts like those with labels 1, **n** is defined as


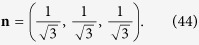


While, on the edges with label 2


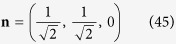


At the bottom corners of the posts with label 3,


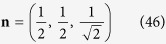


On the edges with labels 4 and 5,


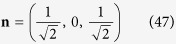


In a same way, the values of **n** at the other corners and edges of the surface can be defined.

## Additional Information

**How to cite this article**: Zu, Y.Q. and Yan, Y.Y. Single Droplet on Micro Square-Post Patterned Surfaces - Theoretical Model and Numerical Simulation. *Sci. Rep.* **6**, 19281; doi: 10.1038/srep19281 (2016).
